# Obesity and the Endometrium: Adipocyte-Secreted Proinflammatory TNF****α**** Cytokine Enhances the Proliferation of Human Endometrial Glandular Cells

**DOI:** 10.1155/2013/368543

**Published:** 2013-10-30

**Authors:** Sangeeta Nair, HoVan Nguyen, Salama Salama, Ayman Al-Hendy

**Affiliations:** ^1^Department of Obstetrics and Gynecology, Center for Women's Health Research, Meharry Medical College, Nashville, TN 37208, USA; ^2^Baylor College of Medicine, 1 Baylor Plaza, Houston, TX 77030, USA

## Abstract

Obesity, a state of chronic inflammation, is associated with poor fertility and low implantation rates and is a well-documented risk factor for endometrial cancer. Adipokines, such as tumor necrosis factor alpha, play an important role in initiation of endometrial cancer. The aim of this study is to evaluate *in vitro* effects of human adipocyte cells (SW872) on growth of endometrial glandular epithelial cells (EGE). *Methods.* We measured cell proliferation and expression of cell-growth proteins—proliferating cell nuclear antigen, cyclin D1, cyclin-dependent kinase-1, and apoptotic markers (BCL-2 and BAK) in human EGE cells cocultured with SW872 cells. EGE cells were also evaluated in SW872-conditioned media neutralized with anti-TNF**α** antibody. *Results.* A significant increase in EGE cell proliferation was observed in both SW872-conditioned media and in coculture (*P* < 0.05). We observed an upregulation of proliferation markers PCNA, cyclin D1, CDK-1, and BCL-2 and decrease in BAK (*P* < 0.05). Neutralization of SW872-conditioned media using anti-TNF**α** antibodies reversed EGE cell proliferation as indicated by BCL-2 expression. *Conclusions.* Adipocytes have potent proliferative paracrine effect on EGE cells which may be, in part, mediated via TNF**α**. Further understanding of the role of obesity in endometrial carcinogenesis should lead to better preventative and therapeutic strategies.

## 1. Introduction

Endometrial cancer is the most commonly diagnosed gynecologic cancer in American women [1]. National Cancer Institute estimates for 2013 indicate 48,560 new cases and 8,000 deaths with endometrial cancer expected in US alone [2]. Endometrial cancer-related mortality is about 14% in Black women compared to White women [3]. Altered sex hormones levels as well as dysregulated levels of cytokines and growth factors are involved in the biology of endometrial cancer [4]. One-third of cancers, including colon, breast, esophagus, and endometrium, are believed to be associated with increase in body weight and low physical activity [5]. Numerous studies have shown high body mass index and obesity to be great risk factors for endometrial cancer [6–8]. 

Adipokines are specific biological factors secreted from the adipose tissue, including several proinflammatory markers such as tumor necrosis factor *α* (TNF-*α*) [9], transforming growth factor-*β* (TGF-*β*) [10], interleukin-6 [11], and monocyte chemoattractant protein-1 [12]. Obesity, a state of low grade chronic systemic inflammation [13], changes the adipokine profiles causing an activation of inflammatory signaling pathways leading to morbid outcomes like tumorigenesis [14, 15]. An increased adipocyte expression and elevated serum level of TNF*α* have been reported in obese rodents and women [15–18]. TNF*α* is a major player in regulation of cell growth, differentiation, inflammation, and metastasis [19]. Inflammatory cytokines are reported to increase cell proliferation and angiogenesis, the hallmarks of tumorigenesis [20]. Although the mechanism involved in the initiation of tumorigenesis via this pathway is not clear, the production of proinflammatory marker TNF*α* locally and/or systemically is believed to play an important role [21, 22]. 

Presence of higher levels of adiposity-related inflammatory cytokines as well as factors such as IL-6, TNF*α*, and C-reactive protein, in addition to lower levels of adiponectin has been implicated as possible contributing factors for initiation and progression of endometrial cancer in women [23]. Interestingly, TNF*α* is also synthesized and secreted from the human endometrial cells [24] and has been associated with physiological and pathological changes in the endometrium-like remodeling, implantation, and cancer [25, 26]. Elevated levels of TNF*α* along with its receptors have been strongly associated with a higher risk of endometrial cancer [27]. In the present study, we investigated the interaction of adipokine-secreting human adipocytes (SW872) with human endometrial glandular epithelial cells (EGE) to better understand the possible biological interplay of obesity and endometrial cancer. The central rationale of this study is that dysregulated adipokine levels secondary to obesity may contribute to the development of endometrial cancer. In this work, we used the *in vitro* cell culture model to investigate cell growth in EGE cells using SW872-conditioned media, and further cocultured the SW872 and EGE cells for additional verification. 

## 2. Methods

### 2.1. Cell Culture

All cell culture experiments including coculture system were performed using human endometrial glandular epithelial cells (EGE, immortalized, nonmalignant human endometrial glandular cells, a generous gift from Dr. Satoru Kyo, Department of Obstetrics & Gynecology, Kanazawa University, Kanazawa, Japan) and human liposarcoma cells (SW872 cells obtained from American Type Culture Collection, Manassas, VA, USA). EGE cells were maintained in DMEM/F12 (1 : 1) supplemented with 10% fetal bovine serum and Insulin Transferrin Selenium (BD biosciences, Bedford, MA, USA) in 5% CO_2_ at 37°C. SW872 cells were maintained in DMEM/F12 with 10% FBS and 1% Penicillin and Streptomycin (Invitrogen, Carlsbad, CA, USA). All coculture experiments were done in 24-well polycarbonate transwell plates with 0.4 *μ*m pore size (Corning, Lowell, MA, USA) as described previously [28].

### 2.2. Colorimetric Assay

EGE cells were cocultured with SW872 cells as well as cultured in SW872-conditioned media and the cell proliferation measured using CyQuant assay, a method based on DNA quantification, as per manufacturer's instructions (Invitrogen, Grand Island, NY, USA). To prepare the conditioned media, SW872 cells were grown to 80% confluence and the media were collected, centrifuged and filtered to remove cell debris. It was diluted 2-fold, 4-fold, and 10-fold using unconditioned media before adding to the EGE cells. 

Briefly, at day 2, day 4, and day 6 of coculture or conditioned media treatment, the culture plates were gently inverted to aspirate the medium from the wells and then washed carefully with PBS. The plates were immediately frozen at −70°C for one hour. The plates were then thawed at room temperature, and 200 *μ*l of CyQuant GR dye/cell lysis buffer was added to each well and mixed gently. The plates were incubated for 5 minutes at room temperature in dark. The sample fluorescence was measured using a fluorescence microplate reader with filters set at 480 nm excitation and 520 nm emission. 

### 2.3. Western Blot

After coculture, the EGE cells were harvested and lysed with a lysis buffer (CelLytic-M, Sigma, St. Louis, MO, USA) containing a protease inhibitor cocktail (Roche Applied Science, Tokyo, Japan). Protein concentration was determined by bicinchoninic acid (BCA) protein assay reagent (Thermo Scientific, Inc., Rockford, IL, USA). The samples were diluted with 4x SDS loading buffer containing *β*-mercaptoethanol. Equal amounts of protein (10 *μ*g) were separated on SDS-polyacrylamide gel electrophoresis and electrotransferred to a polyvinylidene difluoride membrane (Immobilon-P; Millipore Corporation, Bedford, MA, USA). Proteins were detected by immunoblotting followed by ECL chemiluminescence detection (Amersham Biosciences, Piscataway, NJ, USA). Chemiluminescence signals were detected by a luminoimage analyzer SRX-101A (Konica Minolta, Ramsey, NJ, USA). Membranes were immunoblotted with the primary antibody against PCNA (1 : 500), BCL-2 (1 : 500), cyclin D1 (1 : 500), BAK 1 (1 : 500), and CDK-1 (1 : 500) purchased from Santa Cruz Biotechnology (Santa Cruz, CA, USA) and Sigma (St. Louis, MO, USA). After washing, membranes were incubated with horseradish-peroxidase- (HRP-) conjugated secondary antibody (1 : 5000) (Santa Cruz Biotechnology, Santa Cruz, CA, USA). Western blot with anti-*β*-actin antibody (1 : 5,000) was used as loading control. The intensity of each protein band was determined using a scanning densitometer (Alpha Innotech Imager, Santa Clara, CA, USA) and later normalized against the values obtained from *β*-actin. Western blot was also done to determine the expression of BCL-2 in EGE cells after culturing in anti-TNF*α* neutralized conditioned media. For the antibody neutralization experiments, SW872 cells were grown to 80% confluence. The cells were then starved by replacing their medium with FBS-free media and incubation continued for 48 hours. The FBS-free media were then collected, filtered, diluted to 1%, and treated with 1 ng/mL of anti-TNF*α* antibody purchased from R&D systems (Minneapolis, MN, USA) for 1 hour at 37°C. 

### 2.4. Statistical Analysis

All data are presented as means ± standard error (SE) of all values obtained from three to four replicate wells repeated at least three times. Differences between groups were analyzed using Student's *t*-test. *P* ≤ 0.05 was considered statistically significant.

## 3. Results

### 3.1. Enhanced Proliferation of Human Endometrial Glandular Epithelial Cells in Adipocyte-Conditioned Media

**Figure 1:**
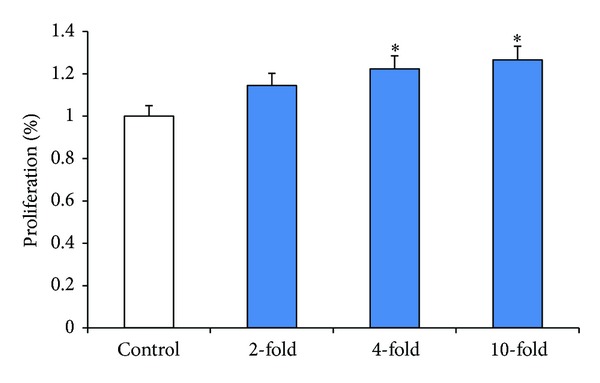
Effect of SW872-conditioned media on proliferation of EGE cells. EGE cells were cultured in 96-well cell culture plate and treated with conditioned media which were diluted from 2- to 10-fold concentrations. Cell proliferation in EGE treated with and without dilutions of SW872-conditioned media was assessed using CyQuant assay as described in Methods. Results are expressed as means ± SE from 3 separate experiments. *Significantly different from the control (*P* < 0.05).

To determine the effect of SW872-conditioned media on EGE cells, SW872 cells were grown to 80% confluence in T200 flasks. The media were collected, diluted to varying concentrations, and added to EGE cells grown to 30% confluence in a 96-well tissue culture plate. Cell proliferation in human endometrial glandular epithelial cells was measured on day 6 using CyQuant cell proliferation kit. Cell proliferation in EGE cells growing with adipocyte-conditioned media at 2-fold, 4-fold, and 10-fold dilutions showed a 14.5%, 22.3%, and 26.65% increase in cell growth, respectively (*P* < 0.05, Figure 1), compared to untreated control. 

### 3.2. Enhanced Proliferation of Human Endometrial Glandular Epithelial Cells When Cocultured with Human Adipocyte Cells

**Figure 2:**
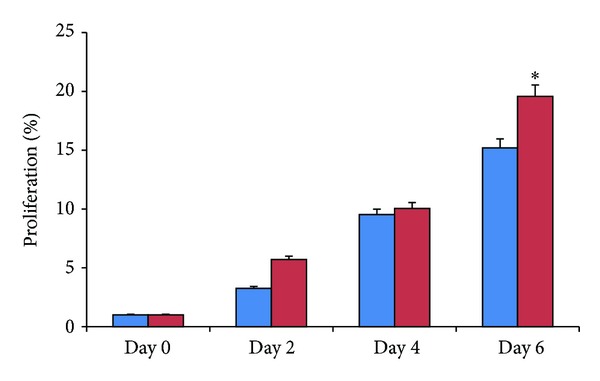
EGE cell proliferation with and without SW872 coculture. EGE cells were cocultured with SW872 cells for days 2, 4, and 6. Comparison in cell proliferation is made with EGE cells grown without SW872 coculture. Proliferation in EGE cells was measured using CyQuant assay. Results are expressed as means ± SE from 3 separate experiments. *Significantly different from the control (*P* < 0.05).

To confirm potential humoral interaction between EGE cells and SW872 cells, cell proliferation was observed in a transwell coculture system (as described in Section 2) without direct cell to cell contact. The control group of EGE cells without SW872 cells coculture was compared with the treatment group of EGE cells and SW872 cells in the transwell coculture system till day 6. An increase in the number of EGE cells was observed with time when cocultured with adipocytes compared to the control. SW872 adipocytes cocultured endometrial cells increased by about 20% on day 6 compared to control cells (*P* < 0.05, Figure 2). 

### 3.3. Adipocyte Coculture Modulates Expression of Protein Markers in Human Endometrial Cells

**Figure 3:**
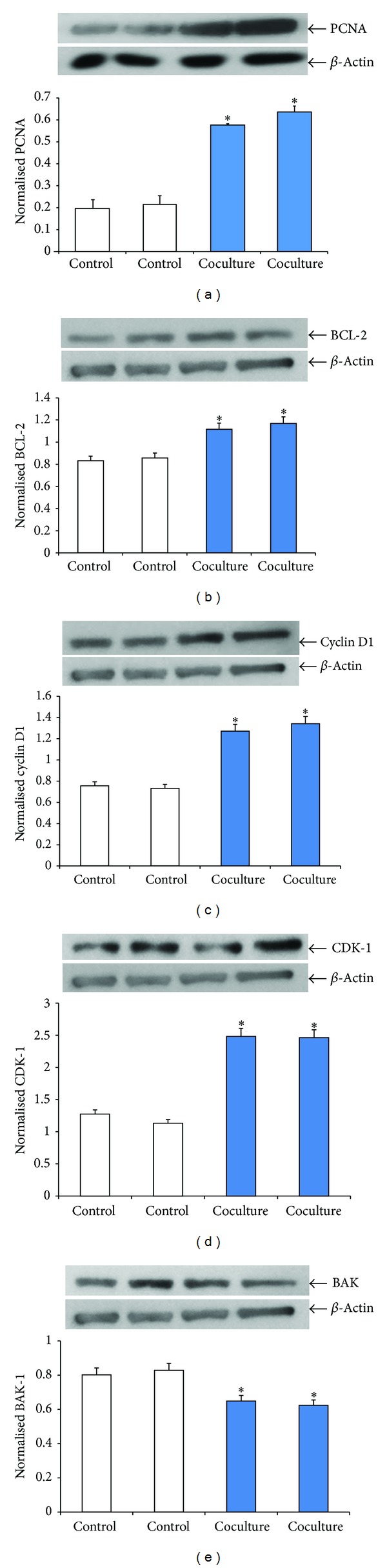
Western blot analysis of PCNA, BCL-2, cyclin D1, CDK-1, and BAK in EGE cells with and without coculture. EGE and SW872 cells were in coculture for 6 days. Lysates prepared from control and cocultured cells were analyzed by Western blotting with (a) anti-PCNA, (b) anti-BCL-2, (c) anticyclin D1, (d) anti-CDK-1, and (e) anti-BAK antibodies. The intensity of each protein signal was quantified and normalized with corresponding *β*-actin. Results shown represent three separate experiments with comparable results. *P < 0.05 compared with control.

Western immunoblot assay showed changes in the expression of various protein markers: cell proliferation (PCNA), anti-apoptosis (BCL-2), cell cycle division (Cyclin D1), cell regulation (CDK-1), and apoptosis marker (BAX). PCNA expression in EGE cells cocultured with SW872 cells showed a significant twofold increase (*P* < 0.05) (Figure 3(a)). Similar significant increases in expression of anti-apoptotic protein marker BCL-2, cyclin D1, and CDK-1 were also recorded (Figures 3(b)–3(d)). The expression of BAK, an apoptotic protein, showed significant induction in control EGE cells compared to cocultured EGE cells (Figure 3(e)). Changes in expression of both cell proliferatory and apoptotic proteins in control versus cocultured EGE cells indicate a positive influence of SW872 cells on the EGE cells to induce proliferatory and inhibit apoptosis markers.

### 3.4. Adipocytes Influence Human Endometrial Cells via Proinflammatory Cytokine TNF-*α*

**Figure 4:**
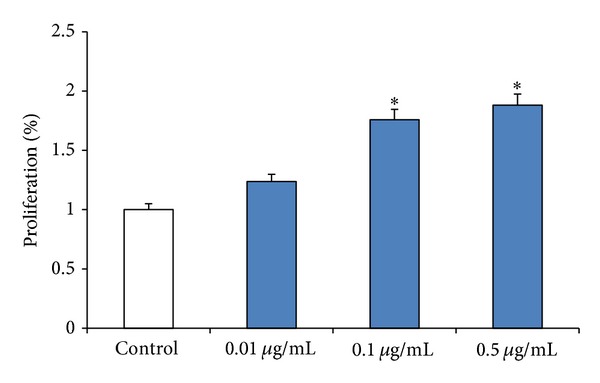
Effect of different concentrations of TNF*α* on EGE cell proliferation. Cell proliferation in EGE cell treated with different concentrations of TNF*α* was assessed using CyQuant assay as described in Methods. Results are expressed as means ± SE from 3 separate experiments. *Significantly different from the control (*P* < 0.05).

**Figure 5:**
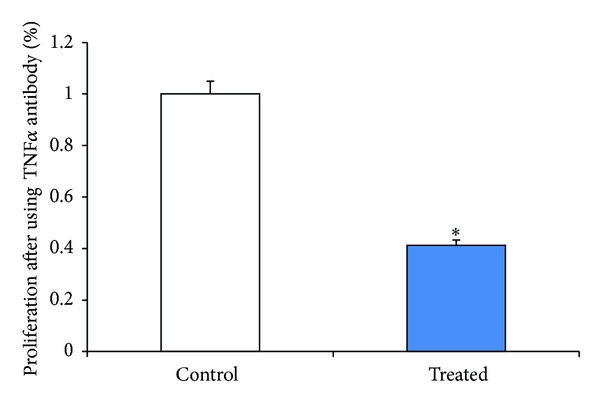
Effects of anti-TNF*α* neutralizing antibodies on EGE cell proliferation. Conditioned media collected from SW872 cells grown to more than 80 percent confluence were centrifuged, filtered, and diluted to 1%. These conditioned media were treated with 1 ng/mL of anti-TNF*α* antibody for an hour at 37°C. The neutralized conditioned media were then added to EGE cells and cell proliferation measured using CyQuant assay. Results shown represent three separate experiments with comparable results. *P < 0.05 (mean ± SE; *n* = 3).

**Figure 6:**
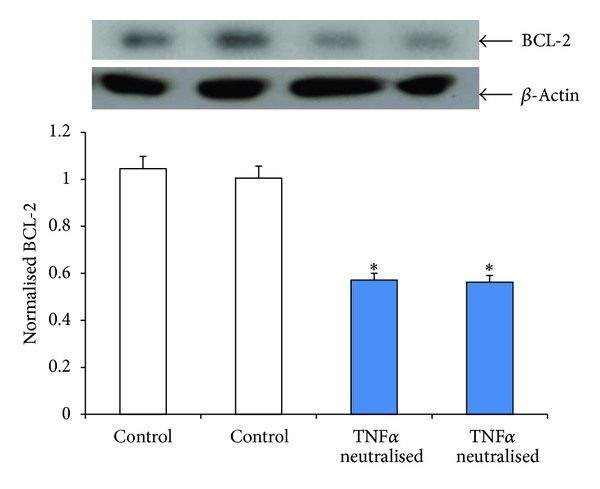
Effects of anti-TNF*α* neutralized conditioned media on the expression of anti-apoptotic BCL-2 in EGE cells. Conditioned media collected from SW872 cells grown to more than 80 percent confluence were centrifuged, filtered, diluted to 1%, and neutralized for an hour with 1 ng/mL of anti-TNF*α* antibody for an hour at 37°C. Lysates prepared from control and treated EGE cells were analyzed by Western blotting with anti-BCL-2 antibody. The intensity of each protein signal was quantified and normalized with corresponding *β*-actin. *P < 0.05 compared with control (mean ± SE; *n* = 3).

Several adipokines have been described in the literature as humoral factors mediating effects of adipocytes (and hence obesity) on various tissues [9, 15, 29, 30] of which TNF*α* is the most widely studied. We consequently evaluated the role of TNF*α* in mediating the proliferatory effects of SW872 adipocytes on EGE cells. Human endometrial glandular epithelial cells were treated with varying concentrations of TNF*α* (0.01, 0.1, and 0.5 *μ*g/mL) for 72 hours and cell proliferation determined using CyQuant assay (Figure 4). We observed that concentrations above 0.1 *μ*g showed a statistically significant increase in cell proliferation compared to control untreated EGE cells (*P* < 0.05). This result suggests that TNF*α* induces dose-dependent glandular endometrial cell proliferation. On the other hand elimination of TNF*α* in the SW872-conditioned media using anti-TNF*α* neutralizing antibody reversed the proliferatory effects in the EGE cells (*P* < 0.05, Figure 5). BCL-2, the antiapoptotic protein, showed reduced expression in cocultured antibody neutralized EGE cells (*P* < 0.05, Figure 6) suggesting the induction of apoptosis.

## 4. Discussion

In this study, we demonstrate that the effects of SW872 cells on EGE cell proliferation are mediated, at least partially, via paracrine effect of TNF*α*. Obesity, an established risk factor for initiation and progression of endometrial cancer, is characterized by a chronic state of inflammation and dysregulated adipokine levels and an activation of inflammatory signaling pathways resulting in pathogenic outcomes including cancers [31–34]. We evaluated the growth stimulatory effect of SW872 cells on EGE cells using a coculture system as an *in vitro* model for cell-cell interaction and demonstrated an increased proliferation of EGE cells. 

Proinflammatory cytokine tumor necrosis factor alpha (TNF*α*) is a multifunctional cytokine shown to activate apoptosis, proliferation, differentiation, and survival responses in several cell types [35]. We also demonstrated that TNF*α* contributes to the EGE cell proliferation in a concentration-dependent manner. EGE cells cocultured with SW872 cells in the transwell system also showed an upregulation of cell proliferatory markers like PCNA, cyclin D-1, CDK-1, and decrease in apoptosis marker BAK. Since these two chambers are physically separate and can communicate only via a membrane (pore size is 0.4 *μ*m), this finding confirms the progrowth stimulatory role of soluble factors secreted from SW872 cells on EGE cell proliferation.

Endometrial cells have been shown to exhibit both receptors of TNF*α* and respond to TNF*α* [24]. In this context we found that neutralization of SW872 cells-conditioned media with anti-TNF*α* antibody reduced proliferation in EGE cells. The expression of anti-apoptosis gene, BCL-2, was downregulated in the EGE cells after treatment with anti-TNF*α* antibody, confirming the role of TNF*α* in an antiapoptotic activation of endometrial cells. Similar antiapoptotic effect of TNF*α* via BCL-2 and nuclear factor kappa *β* (NF*κ*B) pathway have been documented in activated hepatic stellate cells [36]. This indicates that TNF*α* secreted by the SW872 cells increases endometrial cell proliferation, and therefore it is plausible to speculate that TNF*α* secreted from adipose tissue may play an active role in endometrial tumorigenesis *in vivo*. Adipose tissue is reported to secrete TNF*α* both locally and into the circulation [37, 38]. TNF*α* has multiple functions which regulate complex intracellular signaling pathways like NF*κ*B, Akt, p38 MAPK, and others which are well known to transduce TNF*α* signals [39–42]. Our findings suggest a major role of TNF*α* in EGE cell proliferation probably mediated by regulation of downstream proteins involved in inflammatory pathway. Another potential procancerous effect of TNF*α* is via modulating estrogen effects on endometrium. We have recently reported the effects of TNF*α* on estrogen homeostasis and metabolism in endometrial cells including hydroxylation of estrogen to the carcinogenic 4-hydroxy catechol-estrogen which acts as DNA adducts that introduce random DNA mutation and increase DNA instability eventually resulting in cancer initiation and progression [43]. Further studies are needed to identify and characterize adipokines with similar effects on endometrial cells and investigate their mechanism of action.

## 5. Conclusions

Our findings strongly suggest that adipose cells secrete active humoral factors which contribute to endometrial cell proliferation, and a prominent candidate in this panel is the proinflammatory cytokine TNF*α*. Our results are determined from studies conducted in an *in vitro* coculture system which has several limitations like low cell number, finite adipokine volume secreted, and presence of other cytokines/cofactors with possible inhibitory/stimulatory effect. Additional work needs to be carried out to delineate the exact role of this cytokine in endometrial tumorigenesis.
